# Association between smoking status and the prognosis of brain metastasis in patients with non-small cell lung cancer

**DOI:** 10.3389/fonc.2024.1403344

**Published:** 2024-09-19

**Authors:** Xiaofang Zhang, Weilin Zeng, Xingyu Yan, Zheng Wang, Ke Xu, Mo Li, Tianlu Wang, Yingqiu Song

**Affiliations:** ^1^ Department of Radiotherapy, Cancer Hospital of China Medical University, Liaoning Cancer Hospital & Institute, Cancer Hospital of Dalian University of Technology, Shenyang, Liaoning, China; ^2^ The First Clinical College of China Medical University, Shenyang, Liaoning, China; ^3^ Department of Cerebral Surgery, Cancer Hospital of China Medical University, Liaoning Cancer Hospital & Institute, Cancer Hospital of Dalian University of Technology, Shenyang, Liaoning, China; ^4^ Department of Thoracic Surgery, Cancer Hospital of China Medical University, Liaoning Cancer Hospital & Institute, Cancer Hospital of Dalian University of Technology, Shenyang, Liaoning, China

**Keywords:** smoking status, non-small cell lung cancer, brain metastasis, interval time, survival time

## Abstract

**Objective:**

This study aimed to explore the relationship between smoking status and the interval to brain metastasis in patients with non-small cell lung cancer (NSCLC) and its impact on survival time after brain metastasis.

**Methods:**

Data were collected from patients with NSCLC with brain metastases who were treated at our centre between January 2005 and December 2017. Clinical indices such as clinicopathological features and smoking status were recorded, and patients were followed up until 1 September 2022. Based on our inclusion and exclusion criteria, 461 patients were analysed and matched using 1:1 propensity score matching. Three balanced groups were formed: non-smoking (n = 113), smoking cessation (n = 113), and smoking (n = 113). The interval to brain metastasis and overall survival were compared between the groups.

**Results:**

There was a statistically significant difference in the interval to brain metastasis between the non-smoking and smoking cessation groups (*p* = 0.001), as well as between the non-smoking and smoking groups *(p* < 0.001). However, the difference between the smoking cessation and smoking groups was not statistically significant (*p* = 0.106). Multivariate and univariate analyses identified smoking status, clinical stage, lung cancer surgery, chemotherapy, and chest radiotherapy as independent predictors of the interval to brain metastasis. Additionally, the multivariate analysis showed that smoking status, driver gene mutations, and chest radiotherapy independently influenced survival after brain metastasis.

**Conclusion:**

Smoking status in patients with NSCLC affects the interval to brain metastasis and survival after brain metastasis.

## 1 Introduction

Lung cancer is the most common malignancy and a leading cause of cancer-related death worldwide (1), with non-small cell lung cancer (NSCLC) and small cell lung cancer accounting for approximately 85% and 15% of cases, respectively. Approximately 57% of patients with NSCLC have distant metastases at diagnosis, and approximately 20% have brain metastases (BMs) (2, 3). BMs from lung cancer constitute over 50% of all BMs (4). Due to the blood–brain barrier and specific physiological features, treatment strategies for patients with NSCLC-induced BMs are limited, resulting in poor prognosis (5) and a median survival of 4–7 months for untreated patients (6). However, with advances in tumour treatment and diagnostic techniques, patients with NSCLC-induced BMs have shown a median survival of approximately 16 months after treatment (7). This improvement also correlates with increased intracranial progression-free survival and overall survival (OS) (8).

The incidence of BMs is significantly higher in smokers than in non-smokers among patients with NSCLC, indicating a potentially shorter survival (9, 10). A systematic evaluation and meta-analysis of over 10,000 patients with lung cancer across 21 articles published between 1980 and 2021 quantified the impact of smoking cessation at or around the time of diagnosis or during treatment on survival. The results showed that quitting smoking significantly improved the OS in patients with lung cancer, with a particularly greater benefit observed in those with NSCLC (11). Parsons et al. (12) investigated the prognostic impact of smoking cessation on lung cancer by constructing life tables of patients who had quit smoking for several decades. Data obtained from multiple databases indicated that quitting smoking after an early lung cancer diagnosis improved prognosis. The 5-year survival rate for 65-year-old patients with early-stage NSCLC who continued to smoke was 33%, compared to 70% for those who quit (12).

Several studies have confirmed the negative impact of smoking on survival in patients with lung cancer; however, several questions have been raised. Does smoking cessation provide a survival benefit for patients with NSCLC by preventing BM development? How do the interval to BMs and survival after BMs differ among non-smokers, those who quit smoking after diagnosis, and those who continue to smoke after diagnosis? Convincing evidence is urgently needed to answer these questions.

This study aimed to assess the impact of different smoking statuses—never smokers, those who quit smoking after diagnosis, and those who continued to smoke after diagnosis—on the occurrence of BMs and survival after BMs. We retrospectively analysed the timing and prognosis of BMs in patients with NSCLC by identifying the study population, collecting clinical data from a large sample, conducting follow-up observations, obtaining patient survival information, and employing various statistical methods to draw conclusions.

## 2 Materials and methods

### 2.1 Data extraction

This retrospective study included clinical data from patients with NSCLC-induced BMs treated at the Cancer Hospital of China Medical University between January 2005 and December 2017. Patient data at the time of NSCLC diagnosis were collected from the hospital information system, including age, sex, Karnofsky performance status score, smoking status, pathological type, lymph node metastasis, lung cancer site, clinical stage (according to the eighth edition of the TNM staging system published by the International Association for the Study of Lung Cancer), treatment regimen, and treatment-related adverse effects.

### 2.2 Study population

Inclusion criteria were as follows: age ≥ 18 years; a clear pathological NSCLC diagnosis; data regarding the time to diagnosis (via bronchoscopy, lung puncture biopsy, biopsy of metastases, or surgical biopsy); a pathological diagnosis of squamous lung cancer or adenocarcinoma; imaging results (e.g. head-enhanced magnetic resonance imaging and/or pathology) confirming NSCLC BMs; and complete baseline information. Exclusion criteria were as follows: no pathological diagnosis or an unclear pathological type; presence of other primary tumours; incomplete case information; incomplete treatment plan or treatment; unspecified smoking status; and loss to follow-up. Overall, 461 patients were evaluated in the study. All cases were collected before December 2017, and no patients were receiving immunotherapy.

### 2.3 Follow-up visits

The interval to BMs in NSCLC was defined as the period from the date of NSCLC diagnosis to the date of BM diagnosis. The OS for patients with BMs in NSCLC was defined as the period from the date of BM diagnosis to the date of death or the last effective follow-up (the last follow-up cut-off date was 1 September 2022).

### 2.4 Smoking status

Patients were divided into three groups according to their smoking status: non-smoking, smoking cessation, and smoking. According to a study published in the *Journal of Thoracic Oncology* in 2022, ‘quitters’ were defined as individuals who had quit smoking upon or within 3 months after NSCLC diagnosis (11). According to the definition of smoking status by the World Health Organization, the smoking group included individuals who had smoked over 100 cigarettes (including hand-rolled cigarettes, cigars, and cigarillos) in their lifetime and had smoked within 28 d of the evaluation. The smoking cessation group included individuals who had smoked over 100 cigarettes in their lifetime but had not smoked within 28 d of the evaluation. The non-smoking group included individuals who had not smoked more than 100 cigarettes in their lifetime and were currently non-smokers.

### 2.5 Statistical analysis

IBM SPSS (Version 26.0; IBM Corp., Armonk, NY, USA) and R (Version 3.6.3) were used for statistical analyses and visualisation. Two-way comparisons between groups were conducted using 1:1 propensity score matching (PSM) with SPSS software to reduce the effects of bias and confounding variables. The matching variables included sex, T-stage, concurrently diagnosed BMs, and a matching tolerance of 0.1. Optimal performance was achieved by non-relaxation sampling with a randomised case order and a random seed number of six.

Cardinality tests were conducted using the base R package to analyse baseline characteristics before and after PSM. Prognostic correlations were assessed using the Cox proportional hazards regression model with the survival package for R (Version 3.2–10). Survival curves were plotted using the Survminer package for R (Version 0.4.9), and survival data analysis was conducted using the survival package (Version 3.2–10).

## 3 Results

### 3.1 Baseline patient characteristics

**Figure 1:**
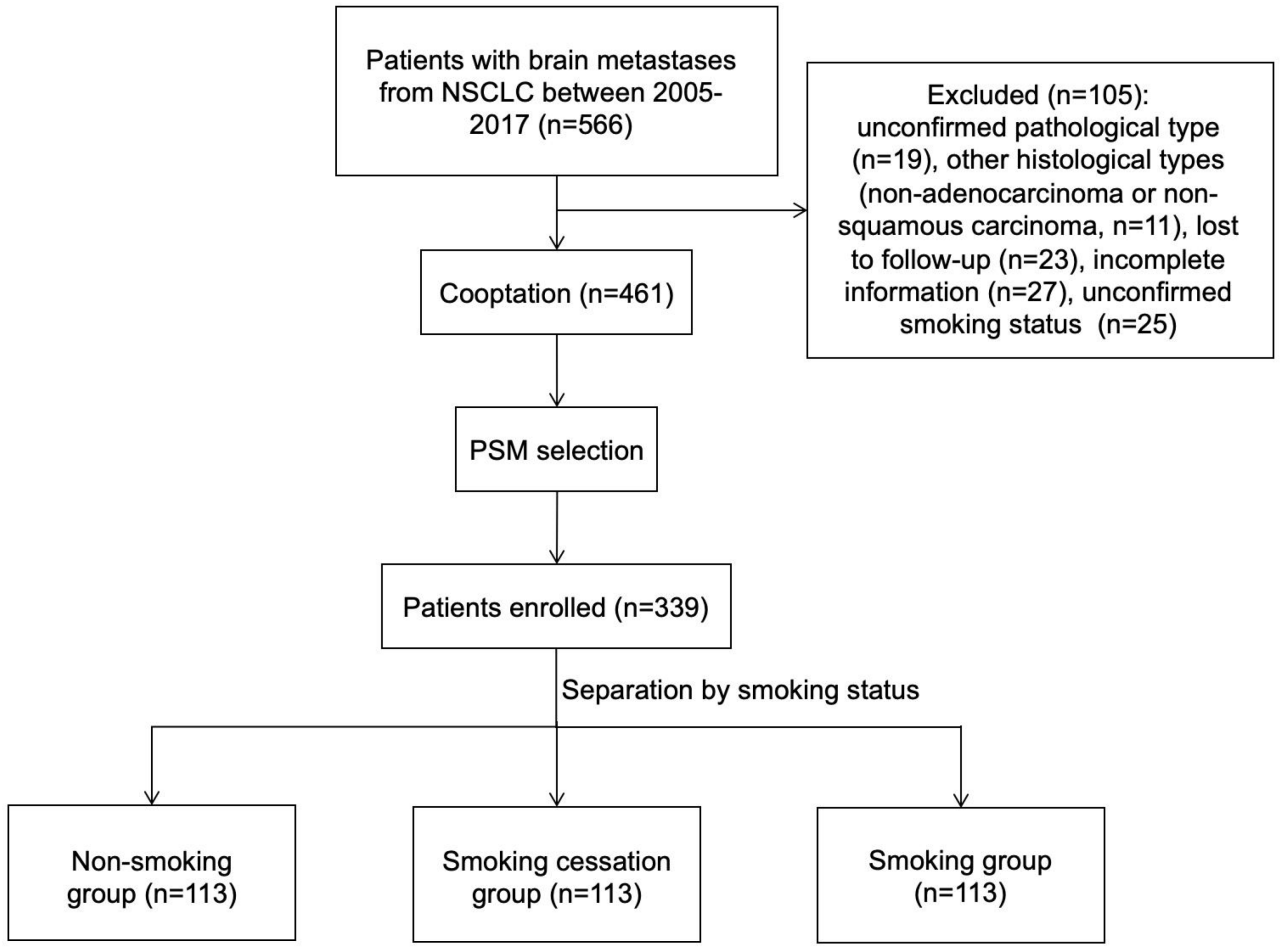
Flow chart for group entry. NSCLC: non-small cell lung cancer.

This study included 566 patients with NSCLC-induced BMs who were first diagnosed at the Cancer Hospital of China Medical University between March 2005 and December 2017. In total, 105 patients were excluded for the following reasons: 19 patients had an unclear pathological diagnosis, 11 had pathological types other than lung adenocarcinoma or squamous lung cancer, 23 were lost to follow-up, 27 had incomplete clinical data, and 25 had unknown smoking status. Ultimately, 461 cases were included in the study. Of these, 164 were non-smokers, 150 were quitters and 147 were current smokers. Using 1:1 PSM, 113 cases each of non-smokers, quitters, and smokers were matched to achieve balance between the groups. The case enrolment process is shown in **Figure 1**.

**Table 1** shows the baseline information of the cases enrolled after PSM. A comparison of each factor among the non-smoking, smoking cessation, and smoking groups revealed no statistical differences between the three groups. To ensure that confounding factors were balanced between each pair of groups, **Supplementary Tables S2**, **S3**, and **S4** present the baseline results of the two-way comparisons between groups after PSM, with no statistical differences in any of the factors between the groups. Univariate and multivariate analysis results of the entire population, baseline results of before/after PSM are shown in **Supplementary Table S1**-**S8**.

**Table 1:** Baseline characteristics of patients with BM at the diagnosis of NSCLC after PSM.

Characteristics	Total (n = 461)	Non-smoking (n = 164)	Smoking cessation (n = 150)	Smoking (n = 147)	χ^2^	*p*-value
Age					0.69	0.710
< 60 years	202	68 (20.1)	70 (20.6)	64 (18.9)		
≥ 60 years	137	45 (13.3)	43 (12.7)	49 (14.5)		
Sex					1.80	0.407
Female	176	64 (18.9)	58 (17.1)	54 (15.9)		
Male	163	49 (14.5)	55 (16.2)	59 (17.4)		
KPS score					0.70	0.706
≥ 90	131	43 (12.7)	47 (13.9)	41 (12.1)		
< 90	208	70 (20.6)	66 (19.5)	72 (21.2)		
Pathological pattern					4.68	0.096
Squamous carcinoma	52	13 (3.8)	24 (7.1)	15 (4.4)		
Adenocarcinoma	287	100 (29.5)	89 (26.3)	98 (28.9)		
Lymph node metastasis					2.37	0.305
No	84	33 (9.7)	28 (8.3)	23 (6.8)		
Yes	255	80 (23.6)	85 (25.1)	90 (26.5)		
Position					0.36	0.834
Peripheral	249	85 (25.1)	81 (23.9)	83 (24.5)		
Central	90	28 (8.3)	32 (9.4)	30 (8.8)		
T classification					0.17	0.916
T1-2	209	71 (20.9)	68 (20.1)	70 (20.6)		
T3-4	130	42 (12.4)	45 (13.3)	43 (12.7)		
N classification					0.47	0.791
N0-1	134	45 (13.3)	42 (12.4)	47 (13.9)		
N2-3	205	68 (20.1)	71 (20.9)	66 (19.5)		
Clinical Stage					0	1.000
I/II/III	129	43 (12.7)	43 (12.7)	43 (12.7)		
IV	210	70 (20.6)	70 (20.6)	70 (20.6)		
Concurrent diagnosis of brain metastases					2.84	0.242
No	244	86 (25.4)	83 (24.5)	75 (22.1)		
Yes	95	27 (8.0)	30 (8.8)	38 (11.2)		
Oncogenic driver mutations					5.15	0.525
Negative	265	86 (25.4)	85 (25.1)	94 (27.7)		
Positive						
EGFR	65	25 (7.4)	23 (6.8)	17 (5.0)		
ALK	8	2 (0.6)	4 (1.2)	2 (0.6)		
KRAS	1	0 (0.0)	1 (0.3)	0 (0.0)		
Surgery					0	1.000
No	261	87 (25.7)	87 (25.7)	87 (25.7)		
Yes	78	26 (7.7)	26 (7.7)	26 (7.7)		
Chemotherapy					2.87	0.238
No	236	72 (21.2)	81 (23.9)	83 (24.5)		
Yes	103	41 (12.1)	32 (9.4)	30 (8.8)		
Thoracic Radiotherapy					0.72	0.698
No	75	23 (6.8)	24 (7.1)	28 (8.3)		
Yes	264	90 (26.5)	89 (26.3)	85 (25.1)		

### 3.2 Analysis of factors influencing the time to the development of brain metastases in NSCLC

**Figure 2:**
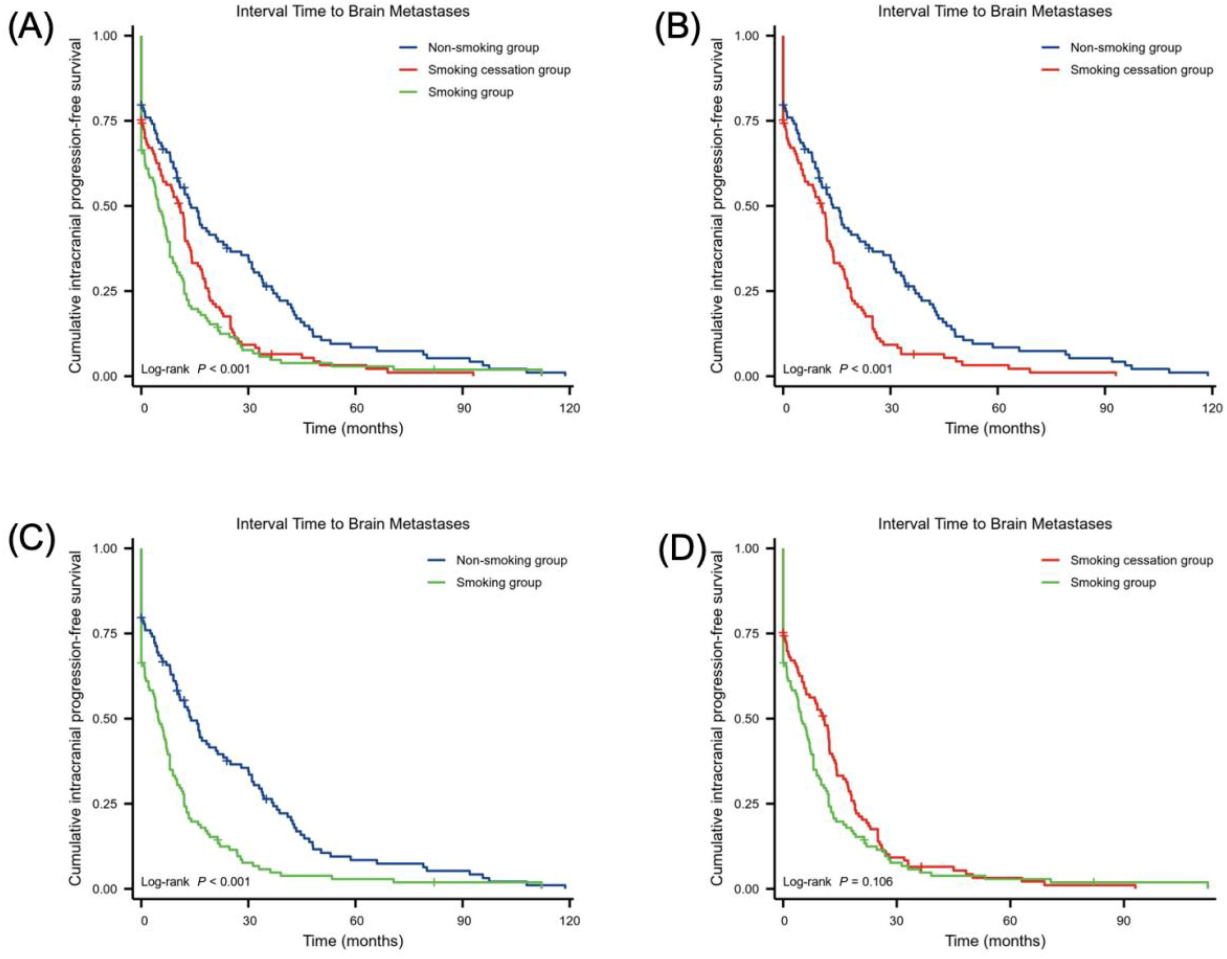
Relationship between smoking status and the interval to BMs in NSCLC. **(A–D)** smoking group (n = 113), non-smoking group (n = 113) and smoking cessation group (n = 113). Statistical analysis was conducted using the log-rank test.

**Figure 2** shows the impact of smoking status on the time to development of BMs in NSCLC. The log-rank test revealed a statistical difference between the non-smoking, smoking cessation, and smoking groups (χ² = 23.46, *p* < 0.001). Comparisons between groups showed that the interval to BMs in NSCLC was longer in the non-smoking group than in the smoking cessation group (χ² =12.05, HR = 1.56 (1.19–2.05), *p* = 0.001). The interval was also longer in the smoking cessation group than in the smoking group (χ² = 20.91, HR = 1.78 (1.35–2.34), *p* < 0.001). However, there was no significant difference in the interval to BMs between the non-smoking and smoking groups (χ² = 2.62, HR = 1.23 (0.94–1.60), *p* = 0.106).

### 3.3 Analysis of factors influencing survival after brain metastases from NSCLC

**Figure 3:**
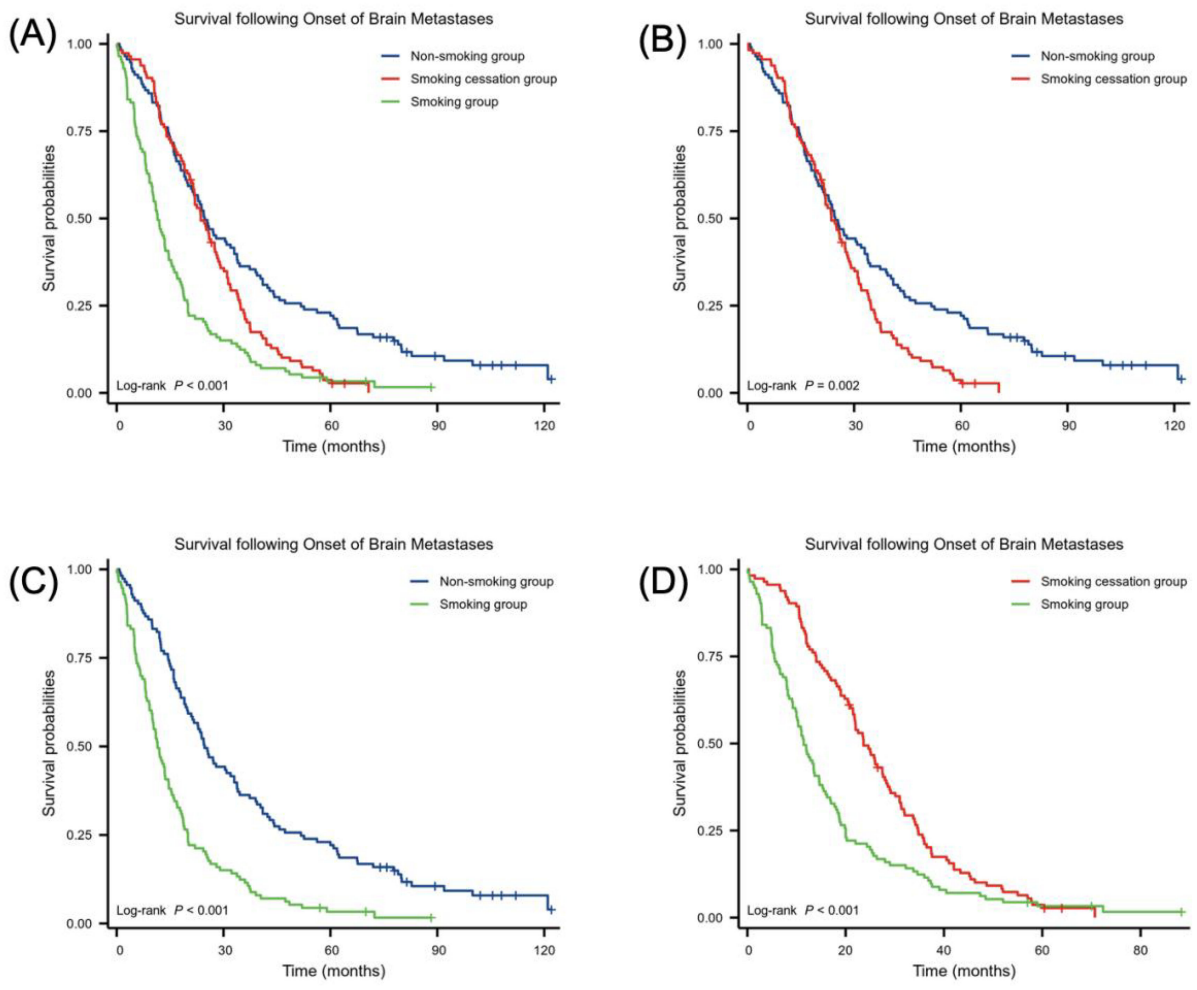
Relationship between smoking status and survival following BM onset. Number of samples in **(A–D)** smoking group (n = 113), non-smoking group (n = 113) and smoking cessation group (n = 113). Statistical analysis was conducted using the log-rank test.

**Figure 3** illustrates the effect of smoking status on survival time following the development of BMs. The log-rank test indicated a significant difference in survival between the non-smoking, smoking cessation, and smoking groups (χ² = 45.78, *p* < 0.001). Comparative analysis using the log-rank test showed that the non-smoking group had a longer survival time after NSCLC BMs than the smoking cessation group, with more non-smokers surviving beyond 60 months. In contrast, survival in the smoking cessation group was more concentrated within 30 months (χ² = 9.18, HR = 1.49 (1.13–1.95), *p* = 0.002). The smoking cessation group had longer survival than the smoking group (χ² = 35.89, HR = 2.17 (1.63–2.89), *p <* 0.001). Additionally, the non-smoking group had longer survival than the smoking group (χ² = 16.15, HR = 1.70 (1.29–2.23), *p <* 0.001.

## 4 Discussion

To assess the impact of smoking cessation on survival in patients with lung cancer, researchers analysed a cohort of patients with cancer who smoked. Using the Cancer Genome Atlas database, they found that smoking cessation was a protective factor for OS in patients with squamous lung cancer, indicating that patients who quit smoking might have longer survival (13). Additionally, Heberg (14) analysed data from 7841 individuals who smoked at the time of lung cancer diagnosis and found a significantly lower mortality risk in those who quit smoking compared to those who continued smoking.

Comparisons of the physical status at 6 and 12 months after lung cancer diagnosis among patients with NSCLC who did and did not quit smoking showed that patients who quit smoking maintained a better physical status (15). One investigator prospectively studied patients with NSCLC recruited between 2007 and 2016 and followed them annually until 2020. The median OS of patients who quit smoking was 21.6 months higher compared to patients who continued smoking. Patients who quit smoking had higher 5-year OS and progression-free survival rates than those who continued to smoke, with smoking cessation linked to a reduced risk of all-cause mortality, cancer-specific mortality, and disease progression (16). Reviews of the relationship between smoking cessation and OS and relapse-free survival among 543 patients with early-stage NSCLC revealed that the hazard ratio decreased with increasing duration of smoking cessation compared to current smokers. Thus, smoking cessation was associated with improved survival in patients with early-stage NSCLC, with longer cessation durations correlating with better survival outcomes (17).

Regarding the effect of smoking on lung cancer–induced BMs, we identified seven relevant articles through a literature search. A retrospective analysis of patient data from these articles revealed that BM incidence was significantly higher in smokers compared to non-smokers, and the progression-free survival and OS of patients with BMs were shorter in smokers (**Supplementary Table S9**). These findings are consistent with our study results.

Studies have shown that smoking is associated with the rapid progression of BMs in patients with lung cancer. This occurs through inflammatory signalling pathways, squamous epithelial chemotaxis–related genes, and glycolysis, leading to oxidative stress and other responses (18). Research indicates that metabolism plays an important role in tumour immunity and that the metabolic phenotype of primary tumour cells differs from that of metastatic tumour cells, making metabolic therapies targeting primary tumours potentially less effective against metastasis (19). Additionally, high metabolic activation was found in MRC1 + CCL18 + M2 macrophages at metastatic sites, and effective neoadjuvant chemotherapy can slow this metabolic activation (20). It has also been reported that neutrophils have complex functions and may play opposite roles in different cancer types, with the neutrophils driving the metastatic niche playing an important role (21). Notably, some studies have demonstrated the ability of nicotine to alter immune cell status, playing a crucial role in the mechanism of BM from lung cancer. Currently, the effects of nicotine on microglia and neutrophils in the brain are the most extensively studied. Specifically, long-term chronic exposure to nicotine in the brain’s pre-metastatic niche causes significant aggregation of N2-neutrophils through the STAT3 pathway. These aggregated N2-neutrophils secrete miR-4466, which promotes the BM of metastatic lung cancer cells through the SKI/SOX2/CPT1A axis (22). Prolonged nicotine exposure also leads to a substantial increase in microglia in the brain, shifting them toward the M2 phenotype. Additionally, M2-microglia enhance IGF-1 and CCL20 secretion and increase SIRPα expression. IGF-1 and CCL20 promote tumour progression, while SIRPα interacts with CD47 expressed on tumour cells to inhibit microglial phagocytosis. This process suppresses the innate immune function of microglia and promotes lung cancer BM. Notably, nicotine enhances this effect (23).

**Figure 4:**
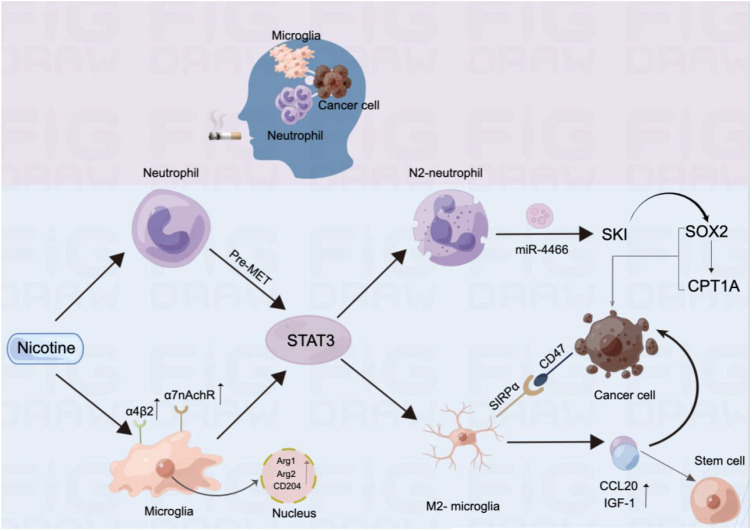
Mechanism by which nicotine promotes BM in lung cancer and alters immune cell infiltration in the brain.

As is shown in **Figure 4**, nicotine promotes tumour growth by activating nicotinic acetylcholine (nACh) receptors in tumour cells. These receptors are expressed in microglia, with the α4β2 receptor being the most abundant nACh receptor in the brain and the main mediator of nicotine dependence (24). Nicotine enhances α7-nACh receptor expression and promotes the M2-type polarisation of microglia by disrupting EGFR signalling and STAT3 pathways, thus promoting cancer cell progression and metastasis. This suggests that nicotine can reprogram the brain tumour microenvironment to promote tumour progression by activating its receptors (25).

Additionally, nicotine in tobacco causes a sharp increase in intracellular reactive oxygen species, which remain at moderate levels during sustained exposure. This abnormal elevation of reactive oxygen species induces the endoplasmic reticulum stress response and activates the unfolded protein response by upregulating binding immunoglobulin protein expression and increasing the phosphorylation level of PERK. Furthermore, prolonged nicotine exposure affects the activation of the p53 protein by sodium arsenite. When p53 is inhibited or damaged, sustained nicotine exposure causes lung epithelial cells to form colonies on soft agar, exhibiting oncogenic properties (26). Researchers from the United States have resolved the cellular heterogeneity of human respiratory epithelial tissue at the single-cell level and comparatively analysed the effects of smoking on individual cell compositions and their intrinsic functions. Their evaluation of the respiratory epithelium found that inflammatory signalling pathways, squamous epithelial chemotaxis–related genes, and glycolysis were significantly upregulated in smokers, while innate immunity and antigen delivery were downregulated. Specifically, pathways significantly upregulated in the mature ciliated cells of smokers included apoptosis regulation, the NOTCH pathway, and the oxidative stress response. Conversely, the expression of genes related to the electron transport chain and lysosomes decreased in mixed-ciliated cells (27).

**Figure 5:**
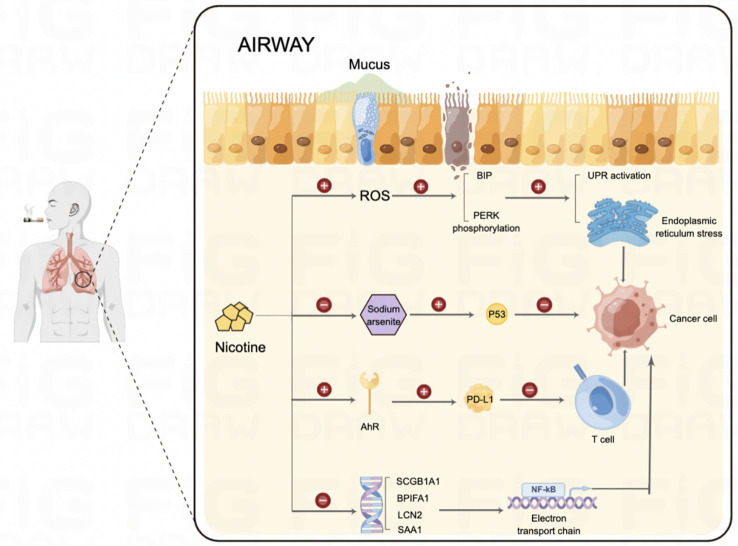
Effects of nicotine on the pulmonary microenvironment.

As is shown in **Figure 5**, Zhou et al. (28) demonstrated that tobacco smoke induces PD-L1 expression in lung epithelial cells through the aromatic hydrocarbon receptor (AhR), enabling the cells to evade T-cell killing and promote tumourigenesis. They also showed that AhR could predict a patient’s response to immunotherapy and be an attractive therapeutic target. Kheradmand et al. (29) found that long-term inhalation of nanoscale carbon black ultrafine particles (15–75 nm) led to mitochondrial damage and metabolic reprogramming of lung macrophages. This reprogramming increases lactate secretion and forms an immunosuppressive microenvironment, ultimately contributing to lung cancer development and metastasis. Huang et al. (30) collected proximal bronchial basal cells from 14 non-smokers and 19 smokers, conducting genome-wide somatic mutation profiling using single-cell multiple displacement amplification. The results showed that the number of mutations in lung cells increased linearly with the years of smoking. However, the increase in cell mutations ceased after 23 years of exposure to smoking factors. This cessation may be related to the body’s enhanced ability to repair DNA damage or detoxify cigarette smoke after long-term exposure.

This study provides clinical evidence that the interval length and prognosis of BMs in patients with NSCLC are significantly associated with smoking status. To eliminate the effects of confounding factors, we equalised baseline differences using PSM. Non-smokers and patients with NSCLC who managed to quit and remain abstinent after diagnosis benefited from clinical care, supporting early smoking cessation as an essential part of lung cancer management and indicating the need for adequate support.

## 5 Conclusion

Smoking status is an independent factor influencing the interval between the onset of BM and prognosis after BM in patients with NSCLC. The median interval lengths for the occurrence of BMs in the non-smoking, smoking cessation, and smoking groups were 12, 10, and 6 months, respectively, with significant differences in the statistical analysis. Independent factors affecting the interval length of BM occurrence in NSCLC included smoking status, clinical stage, lung cancer surgery, chemotherapy, and chest radiotherapy.

The median survival times after BM in the non-smoking, smoking cessation, and smoking groups were 25, 24, and 11 months, respectively, with significant differences in the statistical analysis.

## Data Availability

The original contributions presented in the study are included in the article/**Supplementary Material**. Further inquiries can be directed to the corresponding authors.
