# Supplementary material for: Association between smoking status and the prognosis of brain metastasis in patients with non-small cell lung cancer
Source: Front Oncol. 2024 Sep 19;14:1403344. doi: 10.3389/fonc.2024.1403344 (PMC11446722; doi:10.3389/fonc.2024.1403344)
Supplement: Supplementary file 1 [file DataSheet1.docx]

# Supplementary data

Table S1 shows the baseline information of the included cases before PSM. There were no statistical differences between the two groups in terms of age, KPS score, type of pathology, lymph node metastasis, lung cancer site, N stage, clinical stage, driver mutation, lung cancer surgery, chemotherapy, and chest radiotherapy (*P*＜0.05). There were between-group differences in gender, T-stage, and whether brain metastases were diagnosed at the same time (*P*＜0.05), requiring further equalization of baseline characteristics.

**Table S1.** Baseline characteristics of patients with pre-PSM brain metastases at diagnosis of NSCLC

| **Characteristics** | **Total**  **(n=461)** | **Non-smoking**  **(n=164)** | **Smoking cessation(n=150)** | **Smoking (n=147)** | **χ^2^** | ***P* value** |
| --- | --- | --- | --- | --- | --- | --- |
| **Age** |  |  |  |  | 2.95 | 0.228 |
| ＜60 | 277 | 104 (22.6) | 93 (20.2) | 80 (17.4) |  |  |
| ≥60 | 184 | 60 (13.0) | 57 (12.4) | 67 (14.5) |  |  |
| **Sex** |  |  |  |  | 34.47 | < 0.001 |
| Female | 233 | 113 (24.5) | 59 (12.8) | 61 (13.2) |  |  |
| Male | 228 | 51 (11.1) | 91 (19.7) | 86 (18.7) |  |  |
| **KPS score** |  |  |  |  | 2.05 | 0.358 |
| ≥90 | 184 | 67 (14.5) | 65 (14.1) | 52 (11.3) |  |  |
| ＜90 | 277 | 97 (21.0) | 85 (18.4) | 95 (20.6) |  |  |
| **Pathological pattern** |  |  |  |  | 5.71 | 0.057 |
| Squamous carcinoma | 69 | 17 (3.7) | 30 (6.5) | 22 (4.8) |  |  |
| Adenocarcinoma | 392 | 147 (31.9) | 120 (26.0) | 125 (27.1) |  |  |
| **Lymph node metastasis** |  |  |  |  | 1.29 | 0.525 |
| No | 121 | 48 (10.4) | 38 (8.2) | 35 (7.6) |  |  |
| Yes | 340 | 116 (25.2) | 112 (24.3) | 112 (24.3) |  |  |
| **Position** |  |  |  |  | 4.26 | 0.119 |
| Peripheral | 337 | 129 (28) | 107 (23.2) | 101 (21.9) |  |  |
| Central | 124 | 35 (7.6) | 43 (9.3) | 46 (10.0) |  |  |
| **T classification** |  |  |  |  | 6.51 | 0.039 |
| T1-2 | 282 | 113 (24.5) | 84 (18.2) | 85 (18.4) |  |  |
| T3-4 | 179 | 51 (11.1) | 66 (14.3) | 62 (13.4) |  |  |
| **N classification** |  |  |  |  | 0.27 | 0.875 |
| N0-1 | 195 | 70 (15.2) | 61 (13.2) | 64 (13.9) |  |  |
| N2-3 | 266 | 94 (20.4) | 89 (19.3) | 83 (18.0) |  |  |
| **Clinical stage** |  |  |  |  | 0.39 | 0.823 |
| I/II/III | 163 | 61 (13.2) | 52 (11.3) | 50 (10.8) |  |  |
| IV | 298 | 103 (22.3) | 98 (21.3) | 97 (21.0) |  |  |
| **Concurrent diagnosis of brain metastases** |  |  |  |  | 7.84 | 0.020 |
| No | 315 | 123 (26.7) | 104 (22.6) | 88 (19.1) |  |  |
| Yes | 146 | 41 (8.9) | 46 (10.0) | 58 (12.6) |  |  |
| **Oncogenic driver mutations** |  |  |  |  | 10.52 | 0.396 |
| Negative | 361 | 123(26.7) | 114(24.7) | 124(26.9) |  |  |
| Positive |  |  |  |  |  |  |
| EGFR | 88 | 37 (8.0) | 30 (6.5) | 21 (4.6) |  |  |
| ALK | 9 | 3 (0.7) | 4 (0.9) | 2 (0.4) |  |  |
| KRAS | 1 | 0 (0) | 1 (0.2) | 0 (0) |  |  |
| ERCC1,TUBB3 | 1 | 0 (0) | 1 (0.2) | 0 (0) |  |  |
| MET | 1 | 1 (0.2) | 0 (0) | 0 (0) |  |  |
| **Surgery** |  |  |  |  | 5.90 | 0.052 |
| No | 330 | 107 (23.2) | 109 (23.6) | 114 (24.7) |  |  |
| Yes | 131 | 57 (12.4) | 41 (8.9) | 33 (7.2) |  |  |
| **Chemotherapy** |  |  |  |  | 1.68 | 0.432 |
| No | 120 | 43 (9.3) | 34 (7.4) | 43 (9.3) |  |  |
| Yes | 341 | 121 (26.2) | 116 (25.2) | 104 (22.6) |  |  |
| **Thoracic Radiotherapy** |  |  |  |  | 0.71 | 0.701 |
| No | 364 | 131 (28.4) | 115 (24.9) | 118 (25.6) |  |  |
| Yes | 97 | 33 (7.2) | 35 (7.6) | 29 (6.3) |  |  |

**Note:** Percentages in brackets are rounded to one decimal place, which may result in percentages that add up to 100.1. Same as other Tables in this artical. The number of patients with heterochronous brain metastases with clinical stage I/II/III at initial diagnosis of NSCLC was 163 and 152 with stage IV.

**Table S2.** Baseline characteristics of patients with brain metastases at diagnosis of NSCLC in the non-smoking and smoking cessation groups after PSM

| **Characteristics** | **Total**  **(n=226)** | **Non-smoking**  **(n=113)** | **Smoking cessation(n=113)** | **χ^2^** | ***P* value** |
| --- | --- | --- | --- | --- | --- |
| **Age** |  |  |  | 0.02 | 0.891 |
| ＜60 | 138 | 68 (30.1) | 70 (31) |  |  |
| ≥60 | 88 | 45 (19.9) | 43 (19) |  |  |
| **Sex** |  |  |  | 0.45 | 0.505 |
| Female | 122 | 64 (28.3) | 58 (25.7) |  |  |
| Male | 104 | 49 (21.7) | 55 (24.3) |  |  |
| **KPS score** |  |  |  | 0.17 | 0.684 |
| ≥90 | 90 | 43 (19) | 47 (20.8) |  |  |
| ＜90 | 136 | 70 (31) | 66 (29.2) |  |  |
| **Pathological pattern** |  |  |  | 3.23 | 0.072 |
| Squamous carcinoma | 37 | 13 (5.8) | 24 (10.6) |  |  |
| Adenocarcinoma | 189 | 100 (44.2) | 89 (39.4) |  |  |
| **Lymph node metastasis** |  |  |  | 0.36 | 0.549 |
| No | 61 | 33 (14.6) | 28 (12.4) |  |  |
| Yes | 165 | 80 (35.4) | 85 (37.6) |  |  |
| **Position** |  |  |  | 0.20 | 0.651 |
| Peripheral | 166 | 85 (37.6) | 81 (35.8) |  |  |
| Central | 60 | 28 (12.4) | 32 (14.2) |  |  |
| **T classification** |  |  |  | 0.07 | 0.785 |
| T1-2 | 139 | 71 (31.4) | 68 (30.1) |  |  |
| T3-4 | 87 | 42 (18.6) | 45 (19.9) |  |  |
| **N classification** |  |  |  | 0.07 | 0.785 |
| N0-1 | 87 | 45 (19.9) | 42 (18.6) |  |  |
| N2-3 | 139 | 68 (30.1) | 71 (31.4) |  |  |
| **Clinical Stage** |  |  |  | 0 | 1.000 |
| I/II/III | 86 | 43 (19) | 43 (19) |  |  |
| IV | 140 | 70 (31) | 70 (31) |  |  |
| **Concurrent diagnosis of brain metastases** |  |  |  | 0.09 | 0.759 |
| No | 169 | 86 (38.1) | 83 (36.7) |  |  |
| Yes | 57 | 27 (11.9) | 30 (13.3) |  |  |
| **Oncogenic driver mutations** |  |  |  | 1.76 | 0.625 |
| Negative | 171 | 86 (38.1) | 85 (37.6) |  |  |
| Positive |  |  |  |  |  |
| EGFR | 48 | 25 (11.1) | 23 (10.2) |  |  |
| ALK | 6 | 2 (0.9) | 4 (1.8) |  |  |
| KRAS | 1 | 0 (0.0) | 1 (0.4) |  |  |
| **Surgery** |  |  |  | 0 | 1.000 |
| No | 174 | 87 (38.5) | 87 (38.5) |  |  |
| Yes | 52 | 26 (11.5) | 26 (11.5) |  |  |
| **Chemotherapy** |  |  |  | 1.30 | 0.255 |
| No | 153 | 72 (31.9) | 81 (35.8) |  |  |
| Yes | 73 | 41 (18.1) | 32 (14.2) |  |  |
| **Thoracic Radiotherapy** |  |  |  | 0 | 1.000 |
| No | 47 | 23 (10.2) | 24 (10.6) |  |  |
| Yes | 179 | 90 (39.8) | 89 (39.4) |  |  |

**Table S3.** Baseline characteristics of patients with brain metastases at diagnosis of NSCLC in the non-smoking and smoking groups after PSM

| **Characteristics** | **Total**  **(n=226)** | **Non-smoking**  **(n=113)** | **Smoking**  **(n=113)** | χ**^2^** | ***P* value** |
| --- | --- | --- | --- | --- | --- |
| **Age** |  |  |  | 0.16 | 0.686 |
| ＜60 | 132 | 68 (30.1%) | 64 (28.3%) |  |  |
| ≥60 | 94 | 45 (19.9%) | 49 (21.7%) |  |  |
| **Sex** |  |  |  | 1.44 | 0.231 |
| Female | 118 | 64 (28.3%) | 54 (23.9%) |  |  |
| Male | 108 | 49 (21.7%) | 59 (26.1%) |  |  |
| **KPS score** |  |  |  | 0.02 | 0.891 |
| ≥90 | 142 | 70 (31%) | 72 (31.9%) |  |  |
| ＜90 | 84 | 43 (19%) | 41 (18.1%) |  |  |
| **Pathological pattern** |  |  |  | 0.04 | 0.84 |
| Squamous carcinoma | 28 | 13 (5.8%) | 15 (6.6%) |  |  |
| Adenocarcinoma | 198 | 100 (44.2%) | 98 (43.4%) |  |  |
| **Lymph node metastasis** |  |  |  | 1.92 | 0.166 |
| No | 56 | 33 (14.6%) | 23 (10.2%) |  |  |
| Yes | 170 | 80 (35.4%) | 90 (39.8%) |  |  |
| **Position** |  |  |  | 0.02 | 0.879 |
| Peripheral | 168 | 85 (37.6%) | 83 (36.7%) |  |  |
| Central | 58 | 28 (12.4%) | 30 (13.3%) |  |  |
| **T classification** |  |  |  | 0 | 1 |
| T1-2 | 141 | 71 (31.4%) | 70 (31%) |  |  |
| T3-4 | 85 | 42 (18.6%) | 43 (19%) |  |  |
| **N classification** |  |  |  | 0.02 | 0.892 |
| N0-1 | 92 | 45 (19.9%) | 47 (20.8%) |  |  |
| N2-3 | 134 | 68 (30.1%) | 66 (29.2%) |  |  |
| **Clinical Stage** |  |  |  | 0 | 1 |
| I/II/III | 140 | 70 (31%) | 70 (31%) |  |  |
| IV | 86 | 43 (19%) | 43 (19%) |  |  |
| **Concurrent diagnosis of brain metastases** |  |  |  | 2.16 | 0.142 |
| No | 161 | 86 (38.1%) | 75 (33.2%) |  |  |
| Yes | 65 | 27 (11.9%) | 38 (16.8%) |  |  |
| **Oncogenic driver mutations** |  |  |  | 1.88 | 0.391 |
| Negative | 180 | 86 (38.1) | 94 (41.6) |  |  |
| Positive |  |  |  |  |  |
| EGFR | 42 | 25 (11.1) | 17 (7.5) |  |  |
| ALK | 4 | 2 (0.9) | 2 (0.9) |  |  |
| **Surgery** |  |  |  | 0 | 1 |
| No | 174 | 87 (38.5%) | 87 (38.5%) |  |  |
| Yes | 52 | 26 (11.5%) | 26 (11.5%) |  |  |
| **Chemotherapy** |  |  |  | 2.05 | 0.152 |
| No | 155 | 72 (31.9%) | 83 (36.7%) |  |  |
| Yes | 71 | 41 (18.1%) | 30 (13.3%) |  |  |
| **Thoracic Radiotherapy** |  |  |  | 0.41 | 0.524 |
| No | 51 | 23 (10.2%) | 28 (12.4%) |  |  |
| Yes | 175 | 90 (39.8%) | 85 (37.6%) |  |  |

**Table S4.** Baseline characteristics of patients with brain metastases at diagnosis of NSCLC in the smoking cessation and smoking groups after PSM

| **Characteristics** | **Total**  **(n=226)** | **Smoking cessation(n=113)** | **Smoking**  **(n=113)** | χ**^2^** | ***P* value** |
| --- | --- | --- | --- | --- | --- |
| **Age** |  |  |  | 0.46 | 0.498 |
| ＜60 | 134 | 70 (31) | 64 (28.3) |  |  |
| ≥60 | 92 | 43 (19) | 49 (21.7) |  |  |
| **Sex** |  |  |  | 0.16 | 0.690 |
| Female | 112 | 58 (25.7) | 54 (23.9) |  |  |
| Male | 114 | 55 (24.3) | 59 (26.1) |  |  |
| **KPS score** |  |  |  | 0.47 | 0.495 |
| ≥90 | 88 | 47 (20.8) | 41 (18.1) |  |  |
| ＜90 | 138 | 66 (29.2) | 72 (31.9) |  |  |
| **Pathological pattern** |  |  |  | 1.98 | 0.159 |
| Squamous carcinoma | 39 | 24 (10.6) | 15 (6.6) |  |  |
| Adenocarcinoma | 187 | 89 (39.4) | 98 (43.4) |  |  |
| **Lymph node metastasis** |  |  |  | 0.41 | 0.524 |
| No | 51 | 28 (12.4) | 23 (10.2) |  |  |
| Yes | 175 | 85 (37.6) | 90 (39.8) |  |  |
| **Position** |  |  |  | 0.02 | 0.881 |
| Peripheral | 164 | 81 (35.8) | 83 (36.7) |  |  |
| Central | 62 | 32 (14.2) | 30 (13.3) |  |  |
| **T classification** |  |  |  | 0.02 | 0.891 |
| T1-2 | 138 | 68 (30.1) | 70 (31) |  |  |
| T3-4 | 88 | 45 (19.9) | 43 (19) |  |  |
| **N classification** |  |  |  | 0.30 | 0.586 |
| N0-1 | 89 | 42 (18.6) | 47 (20.8) |  |  |
| N2-3 | 137 | 71 (31.4) | 66 (29.2) |  |  |
| **Clinical Stage** |  |  |  | 0 | 1.000 |
| I/II/III | 86 | 43 (19) | 43 (19) |  |  |
| IV | 140 | 70 (31) | 70 (31) |  |  |
| **Concurrent diagnosis of brain metastases** |  |  |  | 1.03 | 0.310 |
| No | 158 | 83 (36.7) | 75 (33.2) |  |  |
| Yes | 68 | 30 (13.3) | 38 (16.8) |  |  |
| **Oncogenic driver mutations** |  |  |  | 3.02 | 0.389 |
| Negative | 179 | 85 (37.6) | 94 (41.6) |  |  |
| Positive |  |  |  |  |  |
| EGFR | 40 | 23 (10.2) | 17 (7.5) |  |  |
| ALK | 6 | 4 (1.8) | 2 (0.9) |  |  |
| ERCC1,TUBB3 | 1 | 1 (0.4) | 0 (0.0) |  |  |
| **Surgery** |  |  |  | 0 | 1.000 |
| No | 174 | 87 (38.5) | 87 (38.5) |  |  |
| Yes | 52 | 26 (11.5) | 26 (11.5) |  |  |
| **Chemotherapy** |  |  |  | 0.02 | 0.881 |
| No | 164 | 81 (35.8) | 83 (36.7) |  |  |
| Yes | 62 | 32 (14.2) | 30 (13.3) |  |  |
| **Thoracic Radiotherapy** |  |  |  | 0.22 | 0.635 |
| No | 52 | 24 (10.6) | 28 (12.4) |  |  |
| Yes | 174 | 89 (39.4) | 85 (37.6) |  |  |

Table S5 shows the results of the analysis of factors influencing the development of brain metastases in 461 cases of non-small cell NSCLC before PSM and the length of time between the development of brain metastases in NSCLC.

Table S6 shows the results of the analysis of the factors influencing the 339 cases of brain metastases from non-small cell NSCLC after PSM and the median interval length of brain metastases from NSCLC, which was 7 months. The median interval length of time between the occurrence of brain metastases from NSCLC in the non-smoking, smoking cessation and smoking groups was 12, 10 and 6 months, respectively. In the univariate analysis, smoking status (P < 0.001), N stage (P = 0.026), clinical stage (P < 0.001), driver mutation (P = 0.018), lung cancer surgery (P < 0.001), chemotherapy (P < 0.001) and chest radiotherapy (P < 0.001) were all factors influencing the length of interval to brain metastasis from lung cancer. A multifactorial analysis of elements with P values less than 0.1 in the univariate analysis yielded: smoking status (P < 0.001), clinical stage (P < 0.001), lung cancer surgery (P < 0.001), chemotherapy (P < 0.001), and chest radiotherapy (P = 0.006) as independent factors influencing the length of interval between brain metastases from NSCLC.

**Table S5.** Analysis of factors influencing the length of time between the development of brain metastases in NSCLC (pre-PSM)

| **Characteristics** | **n** | **Median length of interval (months)** | **Univariate analysis** | | **Multivariate analysis** | |
| --- | --- | --- | --- | --- | --- | --- |
|  |  |  | HR (95% CI) | ***P* value** | **HR (95% CI)** | ***P* value** |
| **Age** |  |  | 0.984 (0.810-1.195) | 0.869 |  |  |
| ＜60 | 277 | 7 |  |  |  |  |
| ≥60 | 184 | 7 |  |  |  |  |
| **Sex** |  |  | 1.171 (0.968-1.417) | 0.104 |  |  |
| Female | 233 | 7 |  |  |  |  |
| Male | 228 | 7 |  |  |  |  |
| **Smoking status** |  |  | 1.457 (1.296-1.638) | <0.001 | 1.442 (1.280-1.625) | <0.001 |
| Non-smoking | 164 | 11 |  |  |  |  |
| Smoking cessation | 150 | 8 |  |  |  |  |
| Smoking | 147 | 4 |  |  |  |  |
| **KPS score** |  |  | 1.160 (0.954-1.411) | 0.136 |  |  |
| ≥90 | 184 | 5 |  |  |  |  |
| ＜90 | 277 | 7 |  |  |  |  |
| **Pathological pattern** |  |  | 0.846 (0.653-1.096) | 0.206 |  |  |
| Squamous carcinoma | 69 | 6 |  |  |  |  |
| Adenocarcinoma | 392 | 7 |  |  |  |  |
| **Lymph node metastasis** |  |  | 1.120 (0.901-1.391) | 0.308 |  |  |
| No | 121 | 8 |  |  |  |  |
| Yes | 340 | 7 |  |  |  |  |
| **Position** |  |  | 1.178 (0.951-1.459) | 0.134 |  |  |
| Peripheral | 337 | 7 |  |  |  |  |
| Central | 124 | 6 |  |  |  |  |
| **T classification** |  |  | 1.175 (0.965-1.432) | 0.109 |  |  |
| T1-2 | 282 | 7 |  |  |  |  |
| T3-4 | 179 | 6 |  |  |  |  |
| **N classification** |  |  | 1.251 (1.030-1.520) | 0.024 | 1.092 (0.895-1.333) | 0.386 |
| N0-1 | 195 | 8 |  |  |  |  |
| N2-3 | 266 | 5 |  |  |  |  |
| **Clinical Stage** |  |  | 2.214 (1.806-2.713) | <0.001 | 1.539 (1.225-1.933) | <0.001 |
| I/II/III | 163 | 14 |  |  |  |  |
| IV | 298 | 1 |  |  |  |  |
| **Oncogenic driver mutations** |  |  | 1.024(0.808-1.296) | 0.847 |  |  |
| Negative | 361 | 6 |  |  |  |  |
| Positive | 100 | 5 |  |  |  |  |
| EGFR | 88 | 4 |  |  |  |  |
| ALK | 9 | 12 |  |  |  |  |
| KRAS | 1 | 1 |  |  |  |  |
| ERCC1, TUBB3 | 1 | 0 |  |  |  |  |
| MET | 1 | 0 |  |  |  |  |
| **Surgery** |  |  | 0.341 (0.272-0.428) | <0.001 | 0.413 (0.321-0.531) | <0.001 |
| No | 330 | 1 |  |  |  |  |
| Yes | 131 | 17 |  |  |  |  |
| **Chemotherapy** |  |  | 0.579 (0.463-0.723) | <0.001 | 0.540 (0.429-0.679) | <0.001 |
| No | 120 | 1 |  |  |  |  |
| Yes | 341 | 10 |  |  |  |  |
| **Thoracic Radiotherapy** |  |  | 0.608 (0.479-0.772) | <0.001 | 0.700 (0.547-0.897) | 0.005 |
| No | 364 | 5 |  |  |  |  |
| Yes | 97 | 13 |  |  |  |  |

**Table S6.** Analysis of factors influencing the length of time between the development of brain metastases in NSCLC (after PSM)

| **Characteristics** | **n** | **Median length of interval (months)** | **Univariate analysis** | | **Multivariate analysis** | |
| --- | --- | --- | --- | --- | --- | --- |
|  |  |  | HR (95% CI) | ***P* value** | **HR (95% CI)** | ***P* value** |
| **Age** |  |  | 0.962 (0.770-1.202) | 0.731 |  |  |
| ＜60 | 202 | 7 |  |  |  |  |
| ≥60 | 137 | 10 |  |  |  |  |
| **Sex** |  |  | 0.977 (0.785-1.217) | 0.837 |  |  |
| Female | 176 | 7 |  |  |  |  |
| Male | 163 | 9 |  |  |  |  |
| **Smoking status** |  |  | 1.402 (1.224-1.605) | <0.001 | 1.405 (1.223-1.615) | <0.001 |
| Non-smoking | 113 | 12 |  |  |  |  |
| Smoking cessation | 113 | 10 |  |  |  |  |
| Smoking | 113 | 5 |  |  |  |  |
| **KPS score** |  |  | 1.126 (0.898-1.413) | 0.305 |  |  |
| ≥90 | 131 | 9 |  |  |  |  |
| ＜90 | 208 | 8 |  |  |  |  |
| **Pathological pattern** |  |  | 0.880 (0.651-1.190) | 0.406 |  |  |
| Squamous carcinoma | 52 | 6 |  |  |  |  |
| Adenocarcinoma | 287 | 8 |  |  |  |  |
| **Lymph node metastasis** |  |  | 1.210 (0.936-1.566) | 0.146 |  |  |
| No | 84 | 9 |  |  |  |  |
| Yes | 255 | 8 |  |  |  |  |
| **Position** |  |  | 1.078 (0.842-1.380) | 0.551 |  |  |
| Peripheral | 249 | 9 |  |  |  |  |
| Central | 90 | 7 |  |  |  |  |
| **T classification** |  |  | 1.106 (0.880-1.391) | 0.386 |  |  |
| T1-2 | 209 | 8 |  |  |  |  |
| T3-4 | 130 | 8 |  |  |  |  |
| **N classification** |  |  | 1.296 (1.032-1.627) | 0.026 | 1.164 (0.922-1.468) | 0.201 |
| N0-1 | 134 | 9 |  |  |  |  |
| N2-3 | 205 | 8 |  |  |  |  |
| **Clinical Stage** |  |  | 2.224 (1.763-2.807) | <0.001 | 1.591 (1.228-2.062) | <0.001 |
| I/II/III | 129 | 14 |  |  |  |  |
| IV | 210 | 1 |  |  |  |  |
| **Oncogenic driver mutations** |  |  | 1.287 (0.985-1.682) | 0.065 | 1.112 (0.849-1.457) | 0.441 |
| Negative | 265 | 10 |  |  |  |  |
| Positive | 74 | 4 |  |  |  |  |
| EGFR | 65 | 3 |  |  |  |  |
| ALK | 8 | 10 |  |  |  |  |
| ERCC1,  TUBB3 | 1 | 0 |  |  |  |  |
| **Surgery** |  |  | 0.362 (0.280-0.467) | <0.001 | 0.434 (0.327-0.576) | <0.001 |
| No | 261 | 4 |  |  |  |  |
| Yes | 78 | 17 |  |  |  |  |
| **Chemotherapy** |  |  | 0.555 (0.424-0.726) | <0.001 | 0.557 (0.422-0.735) | <0.001 |
| No | 236 | 4 |  |  |  |  |
| Yes | 103 | 16 |  |  |  |  |
| **Thoracic Radiotherapy** |  |  | 0.600 (0.459-0.784) | <0.001 | 0.682 (0.518-0.898) | 0.006 |
| No | 75 | 1 |  |  |  |  |
| Yes | 264 | 10 |  |  |  |  |

Table S7 shows the results of the analysis of factors influencing the length of survival for brain metastases in 461 patients with NSCLC before PSM.

Table S8 shows the results of the analysis of factors influencing the length of survival for brain metastases in 339 patients with non-small cell NSCLC after PSM. 1 and 2 year survival rates were 70.5% and 40.7% respectively, with a median survival time of 20 months. The median length of survival after brain metastases from NSCLC in the non-smoking, smoking cessation and smoking groups was 25, 24 and 11 months, respectively. In the univariate analysis, smoking status (*P*＜0.01), KPS score (*P* = 0.022), type of pathology (*P* = 0.023), site of lung cancer (*P* = 0.044) and chest radiotherapy (*P* = 0.039) were all factors influencing the length of survival after brain metastasis in patients. A multifactorial analysis of elements with P values less than 0.1 in the univariate analysis yielded: smoking status (*P* < 0.001), driver mutation (*P* = 0.030), and chest radiotherapy (*P* = 0.002) as independent factors influencing the length of survival after brain metastasis in NSCLC.

**Table S7.** Analysis of factors influencing prognosis after the development of brain metastases in NSCLC (pre-PSM)

| **Characteristics** | **n** | **Survival rate(%)** | | **Survival probabilities (months)** | **Univariate analysis** | | **Multivariate analysis** | |
| --- | --- | --- | --- | --- | --- | --- | --- | --- |
|  |  | **1-year** | **2-years** |  | HR (95% CI) | ***P* value** | **HR (95% CI)** | ***P* value** |
| **Age** |  |  |  |  | 1.111 (0.915-1.349) | 0.287 |  |  |
| ＜60 | 277 | 70.4 | 43.3 | 20 |  |  |  |  |
| ≥60 | 184 | 71.7 | 38.6 | 20 |  |  |  |  |
| **Sex** |  |  |  |  | 1.347 (1.112-1.632) | 0.002 | 1.109 (0.906-1.359) | 0.316 |
| Female | 233 | 73.0 | 45.5 | 21 |  |  |  |  |
| Male | 228 | 68.9 | 37.3 | 19 |  |  |  |  |
| **Smoking status** |  |  |  |  | 1.742 (1.540-1.971) | <0.001 | 1.752 (1.539-1.994) | <0.001 |
| Non-smoking | 164 | 83.5 | 54.3 | 26 |  |  |  |  |
| Smoking cessation | 150 | 83.3 | 50.0 | 24 |  |  |  |  |
| Smoking | 147 | 44.2 | 18.4 | 10 |  |  |  |  |
| **KPS score** |  |  |  |  | 1.188 (0.978-1.444) | 0.082 | 1.147 (0.942-1.396) | 0.172 |
| ≥90 | 184 | 75.5 | 50.0 | 24 |  |  |  |  |
| ＜90 | 277 | 67.9 | 35.7 | 19 |  |  |  |  |
| **Pathological pattern** |  |  |  |  | 0.632 (0.488-0.820) | <0.001 | 0.649 (0.468-0.898) | 0.009 |
| Squamous carcinoma | 69 | 62.3 | 30.4 | 15 |  |  |  |  |
| Adenocarcinoma | 392 | 72.4 | 43.4 | 21 |  |  |  |  |
| **Lymph node metastasis** |  |  |  |  | 1.031 (0.830-1.281) | 0.781 |  |  |
| No | 121 | 67.8 | 46.3 | 23 |  |  |  |  |
| Yes | 340 | 72.1 | 39.7 | 20 |  |  |  |  |
| **Position** |  |  |  |  | 1.240 (1.002-1.536) | 0.048 | 1.079 (0.832-1.401) | 0.565 |
| Peripheral | 337 | 73.0 | 43.0 | 21 |  |  |  |  |
| Central | 124 | 65.3 | 37.1 | 16 |  |  |  |  |
| **T classification** |  |  |  |  | 1.110 (0.914-1.349) | 0.293 |  |  |
| T1-2 | 282 | 73.4 | 42.9 | 21 |  |  |  |  |
| T3-4 | 179 | 67.0 | 39.1 | 19 |  |  |  |  |
| **N classification** |  |  |  |  | 1.058 (0.873-1.283) | 0.564 |  |  |
| N0-1 | 195 | 69.2 | 47.2 | 23 |  |  |  |  |
| N2-3 | 266 | 72.2 | 37.2 | 19 |  |  |  |  |
| **Clinical Stage** |  |  |  |  | 0.883 (0.724-1.077) | 0.219 |  |  |
| I/II/III | 163 | 69.3 | 40.5 | 21 |  |  |  |  |
| IV | 298 | 71.8 | 41.9 | 20 |  |  |  |  |
| **Concurrent diagnosis of brain metastases** |  |  |  |  | 0.843 (0.687-1.035) | 0.102 |  |  |
| No | 315 | 69.2 | 40.0 | 20 |  |  |  |  |
| Yes | 146 | 74.7 | 44.5 | 22 |  |  |  |  |
| **Oncogenic driver mutations** |  |  |  |  | 0.752(0.873-1.283) | 0.002 | 0.752(0.590-0.960) | 0.022 |
| Negative | 361 | 67.6 | 38.8 | 19 |  |  |  |  |
| Positive | 100 | 82.0 | 51.0 | 25 |  |  |  |  |
| EGFR | 88 | 81.8 | 48.9 | 23 |  |  |  |  |
| ALK | 9 | 100.0 | 77.8 | 35 |  |  |  |  |
| KRAS | 1 | 0.0 | 0.0 | 3 |  |  |  |  |
| ERCC1,  TUBB3 | 1 | 100.0 | 0.0 | 21 |  |  |  |  |
| MET | 1 | 100.0 | 100.0 | 69 |  |  |  |  |
| **Surgery** |  |  |  |  | 0.867 (0.702-1.071) | 0.185 |  |  |
| No | 330 | 70.3 | 39.1 | 19 |  |  |  |  |
| Yes | 131 | 72.5 | 47.3 | 23 |  |  |  |  |
| **Chemotherapy** |  |  |  |  | 1.106 (0.889-1.376) | 0.366 |  |  |
| No | 120 | 70.8 | 42.5 | 21 |  |  |  |  |
| Yes | 341 | 71.0 | 41.1 | 20 |  |  |  |  |
| **Thoracic Radiotherapy** |  |  |  |  | 0.792 (0.626-1.003) | 0.053 | 0.644 (0.503-0.823) | <0.001 |
| No | 364 | 69.0 | 38.2 | 19 |  |  |  |  |
| Yes | 97 | 78.4 | 53.6 | 25 |  |  |  |  |

**Table S8.** Analysis of factors influencing prognosis after the development of brain metastases in NSCLC (after PSM)

| **Characteristics** | **n** | **Survival rate(%)** | | **Survival probabilities (months)** | **Univariate analysis** | | **Multivariate analysis** | |
| --- | --- | --- | --- | --- | --- | --- | --- | --- |
|  |  | **1-year** | **2-years** |  | HR (95% CI) | ***P* value** | **HR (95% CI)** | ***P* value** |
| **Age** |  |  |  |  | 1.081 (0.864-1.351) | 0.496 |  |  |
| ＜60 | 202 | 68.8 | 42.1 | 19 |  |  |  |  |
| ≥60 | 137 | 73.0 | 38.7 | 20 |  |  |  |  |
| **Sex** |  |  |  |  | 1.160 (0.931-1.445) | 0.186 |  |  |
| Female | 176 | 70.5 | 43.2 | 20 |  |  |  |  |
| Male | 163 | 70.6 | 38.0 | 19 |  |  |  |  |
| **Smoking status** |  |  |  |  | 1.602 (1.391-1.846) | <0.001 | 1.671 (1.443-1.935) | <0.001 |
| Non-smoking | 113 | 82.3 | 52.2 | 25 |  |  |  |  |
| Smoking cessation | 113 | 80.5 | 48.7 | 24 |  |  |  |  |
| Smoking | 113 | 48.7 | 21.2 | 11 |  |  |  |  |
| **KPS score** |  |  |  |  | 1.302 (1.038-1.632) | 0.022 | 1.230 (0.974-1.553) | 0.082 |
| ≥90 | 131 | 76.3 | 51.9 | 25 |  |  |  |  |
| ＜90 | 208 | 66.8 | 33.7 | 18 |  |  |  |  |
| **Pathological pattern** |  |  |  |  | 0.707 (0.525-0.953) | 0.023 | 0.767 (0.530-1.110) | 0.159 |
| Squamous carcinoma | 52 | 65.4 | 34.6 | 15 |  |  |  |  |
| Adenocarcinoma | 287 | 71.4 | 41.8 | 20 |  |  |  |  |
| **Lymph node metastasis** |  |  |  |  | 1.137 (0.881-1.468) | 0.324 |  |  |
| No | 84 | 71.4 | 47.6 | 23 |  |  |  |  |
| Yes | 255 | 70.2 | 38.4 | 19 |  |  |  |  |
| **Position** |  |  |  |  | 1.287 (1.006-1.646) | 0.044 | 1.182 (0.873-1.599) | 0.279 |
| Peripheral | 249 | 72.3 | 41.8 | 21 |  |  |  |  |
| Central | 90 | 65.6 | 37.8 | 16 |  |  |  |  |
| **T classification** |  |  |  |  | 1.042 (0.832-1.306) | 0.720 |  |  |
| T1-2 | 209 | 71.8 | 41.6 | 20 |  |  |  |  |
| T3-4 | 130 | 68.5 | 39.2 | 19 |  |  |  |  |
| **N classification** |  |  |  |  | 1.080 (0.863-1.352) | 0.500 |  |  |
| N0-1 | 134 | 69.4 | 45.5 | 21 |  |  |  |  |
| N2-3 | 205 | 71.2 | 37.6 | 19 |  |  |  |  |
| **Clinical Stage** |  |  |  |  | 0.884 (0.705-1.107) | 0.283 |  |  |
| I/II/III | 129 | 68.2 | 39.5 | 18 |  |  |  |  |
| IV | 210 | 71.9 | 41.4 | 20 |  |  |  |  |
| **Concurrent diagnosis of brain metastases** |  |  |  |  | 0.838 (0.656-1.069) | 0.155 |  |  |
| No | 244 | 68.4 | 39.8 | 19 |  |  |  |  |
| Yes | 95 | 75.8 | 43.2 | 20 |  |  |  |  |
| **Oncogenic driver mutations** |  |  |  |  | 0.752 (0.576-0.983) | 0.037 | 0.734 (0.556-0.970) | 0.030 |
| Negative | 265 | 66.8 | 38.5 | 19 |  |  |  |  |
| Positive | 74 | 82.4 | 48.6 | 23 |  |  |  |  |
| EGFR | 65 | 81.5 | 46.2 | 22 |  |  |  |  |
| ALK | 8 | 100.0 | 75.0 | 32 |  |  |  |  |
| ERCC1,  TUBB3 | 1 | 100.0 | 0.0 | 21 |  |  |  |  |
| **Surgery** |  |  |  |  | 0.792 (0.622-1.008) | 0.058 | 0.811 (0.628-1.048) | 0.109 |
| No | 261 | 70.9 | 39.5 | 19 |  |  |  |  |
| Yes | 78 | 69.2 | 44.9 | 22 |  |  |  |  |
| **Chemotherapy** |  |  |  |  | 1.062 (0.815-1.382) | 0.657 |  |  |
| No | 236 | 69.9 | 38.1 | 19 |  |  |  |  |
| Yes | 103 | 71.8 | 46.6 | 23 |  |  |  |  |
| **Thoracic Radiotherapy** |  |  |  |  | 0.757 (0.581-0.986) | 0.039 | 0.651 (0.495-0.857) | 0.002 |
| No | 75 | 62.7 | 36.0 | 20 |  |  |  |  |
| Yes | 264 | 72.7 | 42.0 | 20 |  |  |  |  |

**List of abbreviations**

NSCLC: non-small cell lung cancer

BMs: brain metastases

iPFS: intracranial progression-free survival

OS: overall survival

KPS: karnofsky performance status

PSM: propensity score matching
